# Standard Therapy Plus Adjunctive Vegan Lifestyle Intervention in Metastatic Prostate Cancer: A Case Report

**DOI:** 10.3390/curroncol33080485

**Published:** 2026-08-18

**Authors:** Boštjan Jakše, Zlatko Fras, Anno Graser, Katharina Wirnitzer

**Affiliations:** 1 Independent Researcher, 4280 Kranjska Gora, Slovenia; bostjanjakse@hotmail.com; 2 Division of Medicine, Centre for Preventive Cardiology, University Medical Center, 1000 Ljubljana, Slovenia; 3 Faculty of Medicine, University of Ljubljana, 1000 Ljubljana, Slovenia; 4 Department of Radiology, Ludwig-Maximilians-University, 81377 Munich, Germany; 5 Department of Sport Science, University of Innsbruck, 6020 Innsbruck, Austria; 6 Department of Secondary Education, University College of Teacher Education Tyrol, 6010 Innsbruck, Austria; 7 Department of Pediatric Oncology and Hematology, Otto-Heubner Center for Paediatric and Adolescent Medicine (OHC), Charité–Universitätsmedizin Berlin, 13353 Berlin, Germany; 8 Charité Competence Center for Traditional and Integrative Medicine (CCCTIM), Charité–Universitätsmedizin Berlin, 13353 Berlin, Germany

**Keywords:** prostate cancer, metastasis, androgen deprivation therapy, vegan, plant-predominant, plant-based, prostate-specific antigen, fasting, body composition

## Abstract

Prostate cancer is among the most frequently diagnosed cancers in men globally, with metastatic disease contributing substantially to morbidity and mortality. While conventional therapies remain the standard of care, dietary and lifestyle behaviors are increasingly explored as potentially modifiable factors influencing clinical outcomes, although their role in advanced disease is not well established. We report a 60-year-old, physically active male, lifelong non-smoker without significant comorbidities, diagnosed with de novo metastatic prostate adenocarcinoma (Gleason score 9) and an initial prostate-specific antigen (PSA) of 808.3 µg/L. The patient received standard androgen deprivation therapy. By personal choice, he adopted a whole-food, low-fat (WFLF) vegan diet, structured physical activity, and periodic fasting-mimicking diet cycles. These interventions complemented, but did not replace or delay, conventional treatment. During the 14-month follow-up period, serial PSA measurements, laboratory tests, blood pressure monitoring, dietary assessment, body composition analysis, imaging, and germline genetic testing were performed. PSA declined from 808.3 µg/L to 1.7 µg/L (>99.8%), and remained suppressed throughout follow-up. Concurrent improvements were observed in body composition and metabolic biomarkers. The WFLF vegan diet increased fiber and micronutrient intake while reducing saturated fat and cholesterol. This case demonstrates the successful application and execution of an integrative oncology approach and highlights metastatic prostate cancer managed with standard therapy alongside patient-initiated lifestyle modifications. The findings should be interpreted as descriptive, exploratory, and hypothesis-generating. Given the single-patient design, the independent impact of lifestyle interventions cannot be determined. Controlled studies are warranted to clarify their potential role in advanced prostate cancer management.

## Simple Summary

We report the case of a 60-year-old physically active man with newly diagnosed metastatic prostate cancer (Gleason score 9) and a prostate-specific antigen (PSA) level of 808.3 µg/L. Alongside standard androgen deprivation therapy, he voluntarily adopted a whole-food, low-fat vegan diet, structured multimodal exercise, and periodic fasting-mimicking diets. Over 11 months, PSA declined by more than 99.8% to 1.7 µg/L and remained suppressed, while body composition, metabolic markers, and dietary quality improved. As this is a single-patient case report, the independent contribution of lifestyle changes cannot be determined. Controlled studies are needed to evaluate the potential role of such interventions in advanced prostate cancer management.

## 1. Introduction

Cancer remains a leading cause of death worldwide, causing substantial morbidity and mortality. World Cancer Day 2026 (4 February), under theme “United by Unique,” highlights the need for tailored local responses within a globally unified effort to reduce cancer burden, improve care, and advance equity [1]. Prostate cancer is the second most frequently diagnosed cancer and the fifth leading cause of cancer-related death in men globally, with the highest incidence in the Americas, Europe, Australia, and the Caribbean [2,3]. Although mortality and disability-adjusted life-year rates are declining, absolute case numbers continue to rise due to population ageing and growth [4].

Localized disease is often indolent and managed with active surveillance or definitive local therapy based on risk stratification [5]. In contrast, metastatic disease carries a poor prognosis and requires systemic therapy. Real-world data from Finland indicate that the earlier implementation of novel systemic therapies into routine care for metastatic prostate cancer was associated with improved survival, particularly among patients with metastatic hormone-sensitive disease, in whom median overall survival increased from 32.6 to 54.6 months [6]. Standard first-line treatment is androgen deprivation therapy (ADT), commonly using gonadotropin-releasing hormone (GnRH) antagonists to avoid testosterone flare [7,8], often combined with androgen receptor signalling inhibitors and, in high-volume disease, chemotherapy [9,10].

Despite therapeutic advances, durable complete remissions remain rare, while treatment-related toxicities—including fatigue, sarcopenia, metabolic dysfunction, cognitive impairment, and cardiovascular risk—substantially affect quality of life [11,12,13,14]. These limitations support interest in adjunctive non-pharmacologic interventions.

Plant-predominant diets have been associated with reduced risk of several cancers, including colorectal, breast, pancreatic, and prostate cancer, although effects are heterogeneous [15,16]. Meta-analytic evidence suggests vegetarian and vegan diets are associated with reduced total cancer risk, particularly for gastrointestinal and breast cancers [17]. Extending beyond incidence, higher intake of healthful plant foods correlates with lower prostate cancer progression risk [18,19]. These observations support the broader concept that whole food diets, rather than isolated nutrients, may influence cancer outcomes [20]. Alongside dietary approaches, structured exercise improves cardiorespiratory fitness, preserves muscle mass, and mitigates ADT-related sarcopenia and metabolic toxicity [21,22,23,24]. Similarly, emerging nutritional strategies such as fasting-mimicking diets (FMDs) have shown feasibility and favourable metabolic effects in oncology, including reductions in body weight, waist circumference, and blood pressure, although oncologic endpoints remain under investigation [25,26].

Here, we report a 60-year-old, physically active lifelong non-smoker with high-risk metastatic prostate adenocarcinoma (Gleason 9, cT4N1M1b, prostate-specific antigen (PSA) 808.3 µg/L) treated with standard oncologic therapy alongside comprehensive vegan lifestyle modification, including a WFLF vegan diet, structured aerobic and resistance exercise, and periodic FMD. “Vegan diet” refers to a supplemented WFLF diet excluding all animal-derived products, including fish, shellfish, meat, processed meats, dairy products, eggs, and honey [27,28]. “Vegan lifestyle” encompasses a vegan diet, regular physical activity, avoidance of smoking and substance use, and adequate sleep [29,30,31]. In this case, however, in addition to the patient’s habitual WFLF vegan diet, adherence to a periodic, fully vegan FMD was not practically feasible because small amounts of honey are listed as a minor ingredient, primarily serving as a sweetener that provides some readily available carbohydrates and adds flavour [32,33]. From the perspective of everyday practical relevance, the patient’s adherence to the habitual WFLF vegan diet does not equate to adherence to the FMD, which provides only 0.07% of the patient’s annual calorie intake from honey (details regarding the calculations are provided below in Section 2.5.4. (i)). Therefore, throughout this document, the term “vegan” is used synonymously as an umbrella term to refer to both the “almost vegan” diet (i.e., containing no animal products other than honey) and the associated lifestyle adherence described in this case.

The primary outcome was longitudinal PSA over 11 months with 3-month additional follow-up (14 months total). Secondary outcomes included metabolic and nutritional biomarkers, testosterone, blood pressure, dietary intake, body composition, and imaging-based disease assessment. This case highlights the feasibility and apparent safety of integrative lifestyle approaches alongside standard therapy and underscores the need for prospective trials evaluating their clinical impact.

## 2. Methodological Approach to Treatment and Management of Metastatic Prostate Cancer Patient

### 2.1. Basic Patient Characteristics

A 60-year-old physically active, lifetime non-smoking Caucasian male from Slovenia, with no family history of prostate cancer, had followed a traditional omnivorous diet predominantly based on meat and dairy products, supplemented with multivitamins, omega-3 fatty acids, and dairy- and soy-based protein shakes prepared in dairy milk. His regular physical activity prior to the diagnosis of disease included moderate-intensity resistance training three times weekly and unstructured low-to-moderate-intensity walking. He was referred for urological evaluation following an incidental finding of an extremely elevated PSA, measured on 27 March 2025.

### 2.2. Ethics Approval, Patient Consent, and Data Transparency

Ethical review and approval were waived for this single-patient case report, as it described an individualized clinical care intervention and did not constitute a clinical trial or research intervention requiring institutional review board approval. The study was conducted in accordance with the principles of the Declaration of Helsinki. The patient provided written informed consent for study participation and publication of anonymized clinical and laboratory data (17 December 2025). Copies of all original diagnostic reports were submitted to the journal editorial office for verification, with the patient’s approval. The patient voluntarily provided all materials used for the analyses, including medical records from institutions not involved as co-authors.

### 2.3. Prostate Cancer Treatment Timeline

**Figure 1:**
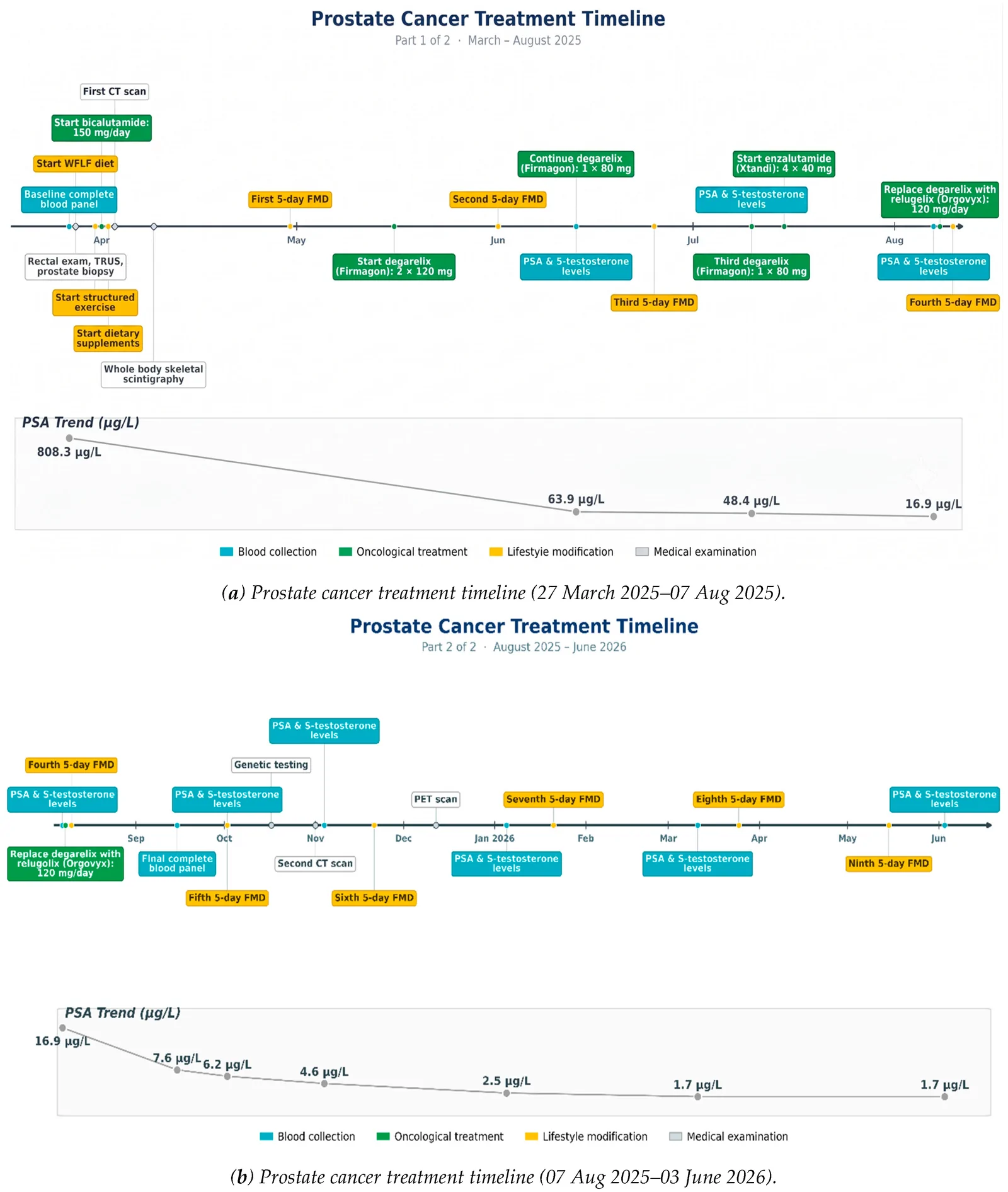
Timeline of prostate cancer treatment timeline for the presented case: (**a**) 27 March–07 August 2025; (**b**) 07 August 2025–03 June 2026.

Figure 1a,b present a detailed treatment timeline for prostate cancer from 27 March 2025 to 3 June 2026, outlining key medical procedures, lifestyle modifications, and oncological treatments.

### 2.4. Initial Diagnostic Workup and Imaging Procedures

Following detection of an elevated PSA (808.3 µg/L) on 27 March 2025, the patient underwent clinical evaluation at the Center for Modern Urology (Ljubljana, Slovenia) on 28 March 2025. Digital rectal examination (DRE) and transrectal ultrasound (TRUS) revealed a heterogeneous, irregularly enlarged prostate with multiple hypoechoic lesions and suspected bladder infiltration. On the same day, a systematic transperineal prostate biopsy was performed under ultrasound guidance using the BK 3000 system with local anaesthesia. Histopathological analysis confirmed prostate adenocarcinoma with a cribriform growth pattern (Gleason score 9 [4+5]). Clinical staging indicated cT4N1M1b disease. The diagnosis of metastatic prostate cancer and the disease stage were established by the treating urologic oncologist and subsequently reviewed and confirmed by the multidisciplinary prostate cancer board at the Institute of Oncology Ljubljana, Slovenia. Initial imaging demonstrated predominantly nodal metastatic involvement, with additional suspicious bone lesions, including a lesion in the left femur, but no extensive confirmed osseous metastatic burden.

In preparation for systemic hormonal therapy, bicalutamide 150 mg daily was initiated on 1 April 2025. The timing of bicalutamide administration relative to degarelix (Firmagon^®^) initiation was determined by the treating urologic oncology team and reflected the clinical assessment of the need for prompt initiation of ADT. Staging investigations, including bone scintigraphy and contrast-enhanced computed tomography (CT) imaging, were performed subsequently according to routine clinical practice and local scheduling; therefore, the complete diagnostic work-up was not available at the time of ADT initiation.

### 2.5. Subsequent Steps and Referral for Further Investigations

#### 2.5.1. Imaging Findings

CT with intravenous contrast performed on 3 April 2025 (MEDILAB d.o.o., Ljubljana, Slovenia) was used for diagnostic assessment and demonstrated a dominant prostatic mass (9 × 5.5 × 5.5 cm) with irregular borders, invading the bladder and seminal vesicles, with extension into the perivesical and perirectal fat. Multiple pathologically enlarged lymph nodes were observed in the pelvic and retroperitoneal regions, including iliac and obturator nodes (up to 30 × 18 mm) and smaller para-aortic nodal metastases. Additional findings included a 15 mm peripheral hepatic hemangioma and a 20 × 10 mm hypodense left adrenal lesion. Follow-up CT of the thoracic and abdominal organs on 29 October 2025 revealed marked regression: prostate volume reduced to 25 mL, near-complete resolution of local infiltration, and regression of enlarged lymph nodes. Positron emission tomography/computed tomography (PET/CT) (performed on 12 December 2025 at the Nuclear Medicine Unit, University Medical Centre, Ljubljana, Slovenia, using a Siemens Biograph mCT system) (Siemens Healthineers, Forchheim, Germany) with 68Ga-PSMA-11, demonstrated uptake in the prostate and abdominal/thoracic lymph nodes, with no bone involvement. The patient continued relugolix (Orgovyx) 120 mg and enzalutamide 160 mg daily, with follow-up planned at 12 weeks.

#### 2.5.2. Skeletal Imaging

Whole-body planar scintigraphy and single-photon emission computed tomography (SPECT) of the thorax and abdomen were performed on 9 April 2025 at the Nuclear Medicine Unit, Slovenj Gradec General Hospital, Ljubljana, Slovenia, using Technescan HDP (647.2 MBq) on a SPECT system (Siemens Symbia Intevo Bold, Siemens Healthineers, Forchheim, Germany). Imaging showed increased osteoblastic activity in both acromioclavicular joints and focal uptake in the distal third of the left femur, raising suspicion of possible osseous metastatic involvement. Mild osteoblastic activity was also observed in the pubic symphysis, left sacroiliac joint, both hip joints, right iliac bone, and the S2–S3 transition zone, predominantly consistent with degenerative changes. Although metastatic involvement of the left femur could not be definitively excluded, no typical scintigraphic features of widespread osteoblastic metastatic disease were identified. The patient was subsequently referred to the Institute of Oncology, Ljubljana, for further evaluation and treatment.

#### 2.5.3. Oncology Evaluation and Systemic Therapy

On 8 May 2025, the patient was evaluated at the Oncology Institute Ljubljana. The case was reviewed by the multidisciplinary urologic oncology board, which established the treatment plan and recommended prompt initiation of systemic therapy. The patient was subsequently referred to the medical oncology clinic for initiation of androgen ADT with the LHRH antagonist degarelix. Degarelix (2 × 120 mg) was administered on 16 May 2025, with interim bicalutamide 150 mg daily. At a follow-up multidisciplinary board meeting on 20 May 2025, the diagnosis of metastatic prostate adenocarcinoma was confirmed, and the treatment plan was reaffirmed. On 13 June 2025, options for systemic treatment intensification were discussed, including the addition of an androgen receptor pathway inhibitor (ARPI) alone or in combination with docetaxel chemotherapy. The option of further systemic treatment intensification was revisited during the medical oncology follow-up on 10 July 2025. After discussion of the available treatment strategies, the patient elected to initiate enzalutamide therapy.

Enzalutamide (Xtandi) 160 mg daily was initiated on 15 July 2025. Nutritional support was offered but declined, and transition from degarelix to relugolix was planned. The patient commenced relugolix 120 mg daily on 8 August 2025, continuing enzalutamide.

**Figure 2:**
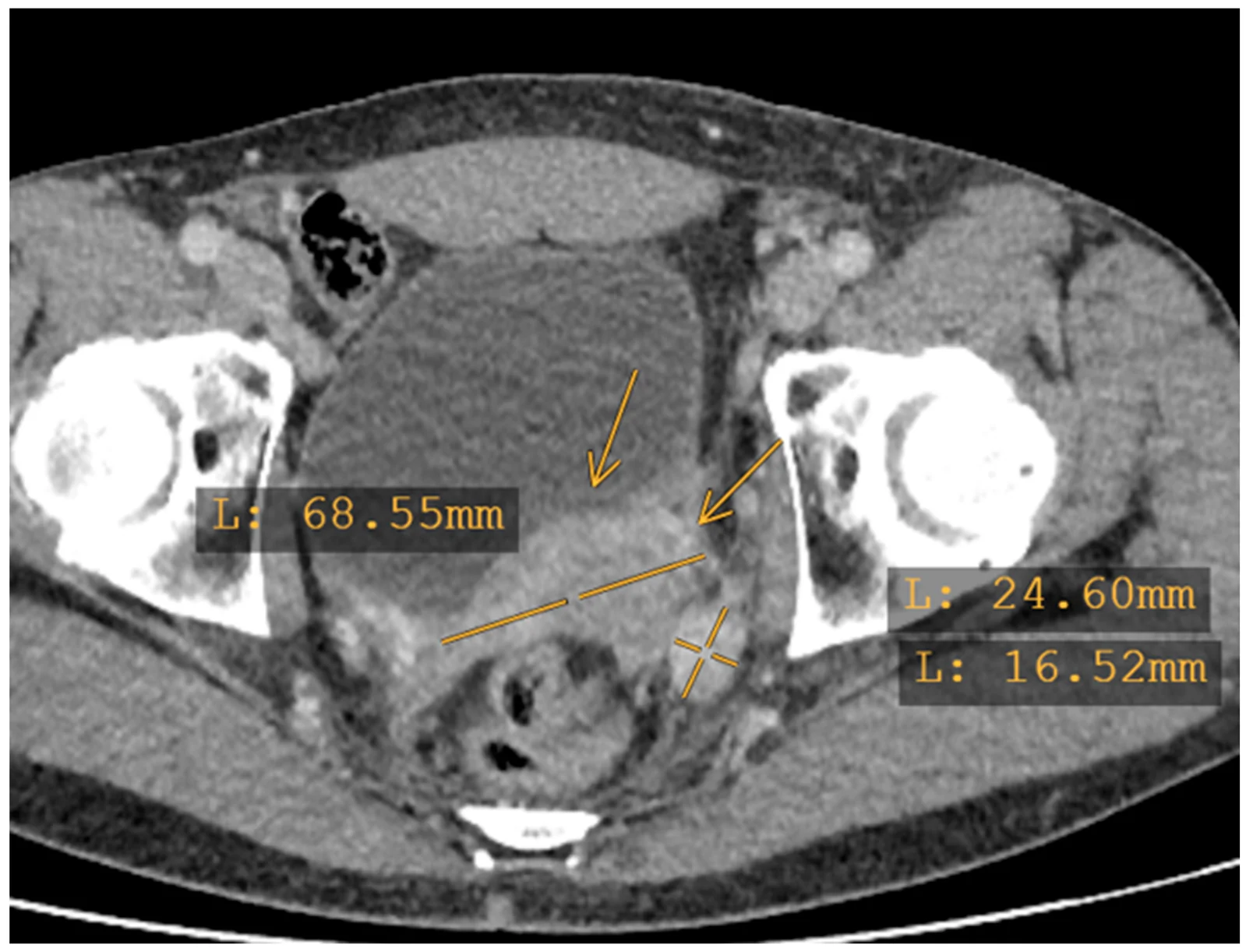
Baseline CT (03 April 2025) showing bilateral, locally advanced, high-grade prostate cancer obliterating the left rectoprostatic angle and infiltrating into the bladder floor (arrows); the image shows the base of the prostate (69 mm in diameter, represented by the diagonal line). A significantly enlarged local lymph node measuring 25 mm × 17 mm (length × width) is visible adjacent to the mesorectal fascia; on PSMA PET, this was confirmed as metastatic involvement. *Note.* The arrows indicate the areas of tumor extension, the measurement lines indicate the corresponding measurements, and “L” denotes the length of the respective measurement.

On 2 October 2025, radiotherapy to the primary tumour was discussed. The patient preferred to defer the decision pending further imaging assessment, including PET/CT, to better define the potential indication for local treatment and weigh its expected benefits against potential risks. Multidisciplinary consultation on 23 October and follow-up assessments on 7 and 19 November 2025 documented the continued decision to defer radiotherapy in the context of ongoing PSA decline, while both radiotherapy and chemotherapy remained potential options for reconsideration should the clinical situation change or disease progression occur. PET/CT was scheduled at the Institute of Oncology Ljubljana for 12 December 2025, with the results presented above (see Section 2.5.1). Figure 2 displays the baseline CT (April 2025).

Molecular genetic testing on 17 October 2025 by the Institute of Oncology Ljubljana Department of Molecular Medicine (Ljubljana, Slovenia) (TruSight Hereditary, MiSeqDx; SeqPilot, JSI Medical Systems, GmbH, Ettenheim, Germany) revealed no germline pathogenic variants per NCCN v2.2026 guidelines.

#### 2.5.4. Lifestyle Change

The patient voluntarily adopted a vegan lifestyle protocol comprising (i) a well-designed WFLF vegan diet, (ii) a structured multimodal exercise programme, and (iii) periodic FMDs.

(i) Well-designed WFLF vegan diet

The patient followed a strictly planned WFLF vegan diet excluding all animal-derived foods, added sugars, refined fats, oils, and unhealthy ultra-processed foods [19,27,28,34,35]. The vegan dietary intervention (except for small amounts of honey contained in the functional nutrition bar included as part of the FMD package, see below) provided the patient with approximately 2500 vegan kcal/day. The dietary plan was designed by a specifically qualified nutritionist (the first author, who holds a PhD in Nutritional Sciences and is certified in WFLF vegan dietary approaches, including eCornell courses in Plant-Based Nutrition and Nutrition for a Healthy Heart). The patient received ongoing nutritional counselling and guidance, including a detailed WFLF dietary plan specifying all meals, recommendations for nutritional supplementation, written dietary guidance, and a recipe book. The intervention was based on a previously described dietary protocol [36,37], with dietary adherence and nutritional adequacy supported and monitored through regular telephone follow-up, individual consultations, dietary questionnaires, selected blood micronutrient measurements, and body composition evaluations throughout the intervention period. The results of these assessments are presented in the Section 3.

As part of the integrative oncology treatment protocol, the patient followed the ProLon FMD program approximately every 1.5 months, corresponding to approximately eight cycles per year. Each 5-day ProLon FMD cycle comprises plant-sourced foods and nutritional products, including vegetable soups, seaweed oil, herbal tea, olives, cabbage biscuits, energy or chocolate bars, and a vegetable supplement formula tablet [38,39]. The package contains nine bars in total, six of which contain small amounts of honey, primarily as a sweetening ingredient that provides some readily available carbohydrates and adds flavour [32,33]. Based on the estimated honey content (10% of a 46-g bar), each honey-containing bar contributed approximately 13 kcal from honey, resulting in an estimated honey-derived energy intake of approximately 78 kcal per cycle and 624 kcal annually [40]. This represents approximately 0.07% of the patient’s estimated annual energy intake. Thus, despite the inclusion of small amounts of honey in the FMD bars, the patient otherwise maintained adherence to a WFLF vegan diet throughout the intervention and to date.

Daily soy intake was encouraged through tofu, tempeh, and soy yogurt. The only higher-fat whole foods permitted were 1.5 tablespoons of ground flaxseeds and 1 tablespoon of unhulled sesame seeds daily; avocados, olives, coconuts, and added fats were excluded. Gluten-containing foods, including mixed-grain bread, were limited to two portions weekly. The diet emphasized deeply pigmented plant foods, including red onions, red cabbage, purple kale, and dark berries, because of their high polyphenol content. Allium and cruciferous vegetables were prepared according to evidence-based recommendations, being chopped and rested for 30–45 min before cooking or processing to maximize bioactive compound availability. The patient consumed one sheet of dried nori seaweed daily (approximately 2 g; ~50 µg iodine total) and one Brazil nut daily (approximately 5 g; 80–100 µg selenium). As he had no previous experience with a WFLF vegan diet, adaptation was required regarding food sourcing, meal preparation, and planning meals away from home. Fluid intake consisted of water and herbal or non-herbal teas, often with turmeric, ginger, or cayenne pepper. Following initiation of enzalutamide (Xtandi), iodised salt intake was maintained and used sparingly, as self-monitored blood pressure remained normal (~110/65 mmHg); flavouring relied primarily on herbs and spices. Apart from reduced social interactions, other lifestyle domains remained unchanged from pre-diagnosis routines.

Supplementation included vitamin B12 (1000 µg cyanocobalamin, three times per week) [27]; vitamin D3 (4000 IU daily in spring/summer, increased to 8000 IU from September due to low UV exposure and limited sun during colder months, aside from recommended outdoor hiking); and a conservative multivitamin mainly for iodine sufficiency. Long-chain omega-3 polyunsaturated fatty acids (eicosapentaenoic acid (EPA) + docosahexaenoic acid (DHA)) were provided at 625 mg/day [28]. An organic, non-soy vegan protein shake (pea, quinoa, flaxseed) contributed ~5% of total energy intake. During colder months, a six-week course of probiotics containing Lactobacillus helveticus and Bifidobacterium lactis supported immune function.

(ii) Multimodal structured exercise regimen

The intervention was developed by two qualified exercise physiology and sports science specialists (the first author, who also has an academic background in Sports Science, including undergraduate specialisation in sports training and strength and conditioning, has approximately 25 years of experience in high-performance sports; and the last author, holding a PhD in Exercise Physiology with a focus on Sports Medicine, and is a qualified Physical Education teacher with a background in sports pedagogy and didactics, with over three decades of experience in providing tailored exercise counselling, guidance, and coaching to physically active individuals and athletes across different performance levels). The regimen incorporated structured resistance training, aerobic exercise, and a morning physical activity routine, aiming to support physical function, preserve lean mass, and maintain metabolic health during ADT.

Therefore, the lifestyle protocol included progressive resistance training (4–5 × /week, ~75 min/session targeting major muscle groups at 6–10 repetition maximum) and interval aerobic uphill walking (5–6 × /week, 10–15 intervals of 1.5 min intense effort, 1 min recovery). Additionally, a daily 30-min morning routine of bodyweight exercises and full-body stretching was performed. No formal meditation or visualization practices were included.

(iii) Periodic FMD

The patient undertook six 5-day FMD cycles on 30 April, 1 June, 25 June, 10 August, 2 October, and 21 November 2025, using a commercially available program (ProLon^®^) [32]. The first three cycles were performed approximately monthly, whereas subsequent cycles were scheduled at approximately six-week intervals following the initiation of enzalutamide therapy. The FMD involved restricted energy and protein intake with fat predominance: ~1000–1100 kcal on day 1 (90% fat) and 700–800 kcal on days 2–5 (40% fat, 10–50% carbohydrates, 2–5% protein) [41]. All other foods and supplements were discontinued during the FMD cycles, with vitamin D3 supplementation maintained only during the winter period. During FMD days, progressive resistance and interval aerobic training were suspended, except for a daily 30-min morning low-intensity bodyweight and stretching routine and shortened high-intensity uphill walking with longer rest.

The standardized FMD protocol is reported for reproducibility; brand and manufacturer names of dietary supplements are omitted. The investigator provided guidance on study design and monitoring but did not supply any intervention components. The treating physician recommended no specific diet or supplements, except vitamin D3 and calcium.

### 2.6. Monitored Results

From 27 March 2025 to 11 March 2026, with an additional 3-month follow-up to 3 June 2026, the primary outcome was PSA, monitored alongside testosterone, to capture disease dynamics during intervals of delayed imaging. During initiation of enzalutamide, daily blood pressure was measured for three months (BM 53, Beurer GmbH, Ulm, Germany) [42] with a representative 20-day every-other-day interval analyzed, reflecting stable values throughout.

Baseline and follow-up venous blood samples (27 March and 15 September 2025) were collected after overnight fasting at Synevo Adria Lab d.o.o., Ljubljana, Slovenia, assessing PSA, metabolic, hepatic, renal, haematological, hormonal, and nutritional markers (apolipoprotein B, glycated haemoglobin (HbA1c), vitamin B12 (cobalamin), 25-hydroxyvitamin D (25[OH]D), folate, iron, ferritin, total testosterone), considered secondary outcomes.

Dietary intake and body composition were continuously monitored. Intake (energy, macronutrients, selected micronutrients, alcohol, water) was assessed via paper food frequency questionnaire (FFQ) at baseline and during intervention [43,44]; nutrient content was calculated using standard portion sizes (National Institute of Public Health, Slovenia) [45] and the OPEN Platform for Clinical Nutrition (Jožef Stefan Institute, Slovenia) [46]. Dietary supplements were recorded separately. Body composition was measured with multi-frequency bioelectrical impedance analysis (Tanita BC-545N) [47] before and after each FMD cycle and within several days post-cycle to capture acute and cumulative effects.

Patient-reported outcomes, including overall well-being, adherence, perceived energy, treatment side effects, effects of diet and FMD, physical strength, and psychological state were assessed via regular in-person and telephone interviews conducted by the first author.

## 3. Results

### 3.1. Longitudinal Changes in Serum PSA and Testosterone Levels During Combined Treatment

Results (Table 1) show a marked reduction in serum PSA and testosterone. PSA decreased progressively at each time point, with an early reduction of up to 92% within approximately 11 weeks (from 27 March to 13 June 2025), followed by a total decline of ~99.8% from 808.3 µg/L to 1.7 µg/L by the end of the intervention. PSA remained suppressed at the 3-month follow-up. Testosterone showed an early marked decline with subsequent fluctuations and stabilization at lower levels later in the course.

### 3.2. Blood Pressure Monitoring During Enzalutamide Initiation

Blood pressure (Table 2) remained stable during enzalutamide initiation, with systolic values ranging 108–116 mmHg and diastolic values 65–73 mmHg, without clinically relevant increases.

### 3.3. Metabolic, Nutritional, Hemodynamic, and Body Composition Changes

Table 3 shows modest reductions in total cholesterol, triglycerides, fasting glucose, and HbA1c. Testosterone decreased substantially, while cortisol increased. Hepatic and renal parameters (AST, ALT, γ-GT, bilirubin, creatinine, eGFR) remained within normal ranges. Uric acid decreased, while haematological indices showed minimal variation. Body composition analysis demonstrated reduced body mass and fat percentage, with minimal loss of fat-free mass.

### 3.4. Body Composition Monitoring During a Representative FMD Cycle

Across repeated 5-day FMD cycles, transient reductions in body mass and fat-free mass occurred, with modest fat loss. Values returned close to baseline within five days post-intervention. Table 4 summarizes these cyclic changes.

### 3.5. Dietary Changes During Treatment

Table 5 shows a shift from the baseline omnivorous diet to a WFLF vegan diet, with increased intake of carbohydrates, fibre, vitamins, and minerals (folate, magnesium, potassium), and reduced saturated fat and cholesterol. Intake of free sugars and higher-fat components was markedly reduced.

### 3.6. Patient-Reported Outcomes

**Figure 3:**
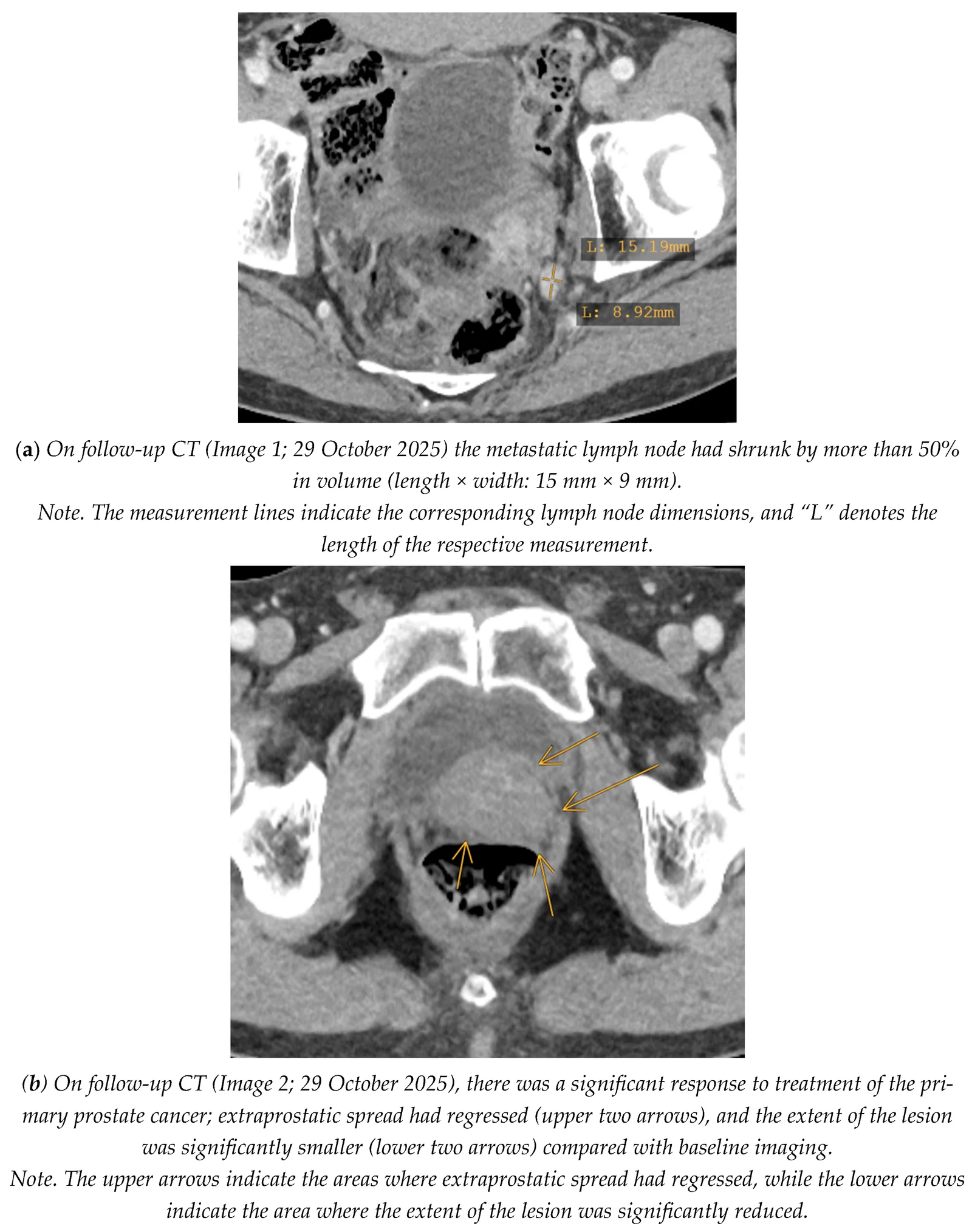
Follow-up CT of prostate cancer imaging (29 October 2025) shows marked responses to the multi-interventional integrative oncological treatment. (**a**) Metastatic lymph node; (**b**) extraprostatic spread and lesion extent.

Patient-reported outcomes indicated early reductions in morning energy and prolonged post-exertional fatigue, with initial adaptation challenges related to meal preparation, food logistics, and reduced palatability compared with the baseline diet. Over time, adaptation improved with greater dietary routine, efficiency in food preparation, and adherence. Nocturia increased initially when fluid intake was not time-restricted (up to ~4 nocturnal voids), but decreased to 1–2 nightly episodes after evening fluid restriction and earlier final meals (~7:30 pm). Figure 3a,b presents the follow-up CT prostate cancer imaging obtained 7 months later (October 2025), together showing pronounced responses to the multi-interventional integrative oncological treatment.

## 4. Discussion

This case describes a 60-year-old physically active, lifelong non-smoker with metastatic prostate adenocarcinoma (Gleason 9; cT4N1M1a/b) and markedly elevated PSA (808 µg/L). Standard ADT, including bicalutamide, GnRH antagonists, and enzalutamide, was combined with a structured WFLF vegan lifestyle, incorporating periodic 5-day FMDs and multimodal exercise. Key outcomes included: (1) PSA reduction from 808.3 µg/L to 1.7 µg/L (99.8%) over 15 months, sustained at 3-month follow-up; (2) modest decreases in total cholesterol, triglycerides, fasting glucose, and HbA1c; (3) improved dietary quality; (4) stable blood pressure and body composition despite enzalutamide initiation and FMD cycles. Testosterone declined early and stabilized at lower levels, while hepatic, renal, and hematologic parameters remained within normal ranges.

ADT is known to reduce PSA and improve progression-free survival [48,49,50]. In this case, the marked PSA decline is most likely attributable to the initiation of ADT. The concurrent favourable metabolic and body composition changes observed during intensive lifestyle intervention represent additional clinically relevant outcomes. While preclinical and observational evidence suggests that dietary and exercise interventions may influence inflammation, metabolic health, and tumour-related pathways, their potential contribution to oncological outcomes in patients receiving ADT remains uncertain and requires further investigation. These findings are consistent with real-world evidence suggesting improved outcomes in metastatic prostate cancer with earlier and more intensive use of modern systemic therapies, including substantial (≈60%) improvements in survival in metastatic hormone-sensitive disease [6]. Although causality cannot be inferred, the observed improvements in metabolic parameters and body composition are consistent with previous reports suggesting potential benefits of well-designed WFLF vegan diet, FMD interventions, and structured exercise as supportive strategies during ADT [20,24,26,51,52,53,54,55,56,57,58,59]. However, the feasibility and potential applicability of such intensive lifestyle interventions may vary according to disease burden, symptom burden, and functional status, particularly in patients with extensive osseous metastatic disease.

Epidemiological data suggest lower prostate cancer risk in vegetarians (−12%) [16], and vegan diets show similar associations [16], potentially mediated by reduced animal protein and insulin-like growth factor 1 signalling. The International Agency for Research on Cancer classifies processed meat as carcinogenic (Group 1) and red meat as probably carcinogenic (Group 2A) [60], while umbrella reviews of systematic reviews and meta-analyses report dose-dependent associations between processed meat consumption and increased risks of gastric, colorectal, and prostate cancers, among other outcomes [61]. Consistently, large prospective cohort data from the European Prospective Investigation into Cancer and Nutrition (EPIC) study have also demonstrated positive associations between processed meat intake and prostate cancer risk, in addition to gastric and oesophageal adenocarcinomas [62].

Adventist cohort data also indicate reduced prostate cancer risk among younger vegans [15]. Randomised and observational studies suggest lifestyle interventions may slow progression and improve quality of life [20,34,35,63,64,65,66]. The Ornish trial demonstrated that WFLF vegan diets, when additionally supplemented with exercise and stress management, can slow early prostate cancer progression [20,34,67], supporting biological plausibility beyond localised disease. The rationale for a WFLF vegan diet in prostate cancer is based on previous clinical and translational research investigating low-fat vegan dietary interventions in men with prostate cancer [20,68,69]. These interventions have been associated with favourable changes in dietary composition, including increased intake of fibre and phytochemicals and reduced intake of saturated fat and cholesterol, as well as changes in selected tumour-related and metabolic parameters [20,68,69,70]. Emerging literature has described less comprehensive personalized dietary approaches in patients with advanced prostate cancer undergoing radiotherapy and radical prostatectomy, with reported improvements in PSA and metabolic parameters and no evidence of recurrence in individual cases [71]. However, these interventions differ substantially in scope and intensity from the present multimodal approach. Recent syntheses further highlight diets rich in plant-sourced fats, soy, cruciferous vegetables, and complex carbohydrates as potentially beneficial in prostate cancer outcomes [72,73]. A meta-analysis of 17 studies reports reduced total cancer risk with vegetarian (−13%) and vegan diets (−23%), including reductions in pancreatic (−23%) and gastric (−45%) cancers [17]. Recent evidence highlights the potential role of diet in prostate cancer outcomes. Souroujon-Torun et al. (2026) reviewed trials and observational studies, showing that a plant-predominant diet, rich in complex carbohydrates, soy, and cruciferous vegetables—while limiting sugars, red and processed meats, and saturated fats—may improve prognosis and reduce mortality [72]. Yet, gaps remain in oncology nutrition training and practice, underscoring the need for enhanced education, access to dietetic support, and robust evidence to integrate dietary strategies into standard care [73].

This case suggests feasibility and safety of integrating intensive lifestyle interventions with ADT. Blood pressure remained stable during enzalutamide therapy, WFLF vegan diet, structured exercise, and periodic FMD cycles, suggesting a potential counterbalancing effect. Patient-reported outcomes showed early adaptation challenges followed by improved adherence and functional routine; nocturia improved from ~4 to 1–2 episodes following fluid timing modification. During repeated 5-day FMD cycles, transient reductions in body mass and fat-free mass were observed, with values returning close to baseline after refeeding. These short-term fluctuations likely reflect physiological changes related to reduced glycogen stores, associated water shifts, and lower gastrointestinal content rather than clinically meaningful loss of lean tissue [38,39]. Nevertheless, ongoing body composition monitoring remains important in patients receiving ADT, given the risk of adverse changes in muscle mass and metabolic health.

Adjunctive low-risk interventions (e.g., mushrooms, creatine, coffee, green tea) may offer supportive metabolic or functional benefits, although evidence remains limited [74,75,76,77,78,79]. Periodic FMD cycles (1.5–3 months) may be implemented to optimise clinical outcomes and potentially slow disease progression, with the option of two consecutive cycles to enhance autophagy. Exercise prescriptions may incorporate planned variation in training volume and intensity, combining uphill interval walking and resistance training, to limit physiological adaptation and sustain metabolic responsiveness over time. PSA remains central for longitudinal monitoring during systemic treatment, complemented by imaging modalities (CT/MRI/PET) for disease assessment [80,81,82]. In this case, PSA dynamics were interpreted together with testosterone suppression, imaging findings, and clinical status during combined systemic treatment and lifestyle intervention. Continued follow-up is required to assess the durability of the observed response, with further management guided by standard oncological evaluation [83].

Beyond objective measurements, patient-reported outcomes provided additional insight into the feasibility and practical implementation of the intervention. The patient experienced initial challenges during dietary transition; however, adherence progressively improved, allowing incorporation of the WFLF vegan diet and lifestyle changes into daily routines. Improvement in nocturia from approximately four to one to two episodes per night following modification of fluid timing further illustrates the potential relevance of individualized lifestyle adjustments. These observations highlight the importance of considering patient needs, social context, and quality-of-life aspects during ADT, including sexual function and management of treatment-related adverse effects. Individualized supportive strategies, including approaches for erectile dysfunction when clinically indicated, should be considered according to patient preferences and clinical circumstances [84].

While these patient-reported observations provide additional context regarding the feasibility of an individualized lifestyle approach during ADT, they should be interpreted within the limitations inherent to a single-case report. Limitations include the single-case design, simultaneous multimodal interventions, and absence of controls, which preclude causal inference. The 92% PSA reduction observed within 2.5 months occurred after initiation of standard ADT with bicalutamide and degarelix, while the lifestyle intervention was implemented concurrently. Therefore, the relative contribution of individual treatment components cannot be determined. The findings should be interpreted as an observation of the clinical course during combined treatment rather than evidence of an independent effect of lifestyle modification on PSA dynamics. A further limitation is the currently available duration and density of longitudinal assessments, as follow-up remains ongoing. To date, comprehensive blood testing and dietary intake assessments have been performed at baseline and again after approximately 6 months, while body composition has been monitored serially throughout the intervention. Further assessments are scheduled as follow-up progresses, including a third CT examination at approximately 6 months and a second PET examination at approximately 10 months, together with subsequent comprehensive blood laboratory and nutritional assessments. The available data, including favourable changes in body composition, consistently very good patient-reported well-being, and serial PSA and testosterone measurements, provide an informative interim longitudinal profile; continued follow-up will allow the durability and clinical course of these observations to be assessed over a longer period.

Overall, this case supports the feasibility and tolerability of integrating a patient-centred, structured lifestyle intervention with ADT in a highly motivated patient with metastatic prostate cancer. While causal effects on oncological outcomes cannot be determined, this approach may represent a supportive strategy warranting further investigation in controlled studies.

## 5. Conclusions

This case illustrates the feasibility of integrating an intensive, patient-centered WFLF vegan diet, structured exercise, and periodic FMD cycles with standard systemic therapy in a highly motivated patient with metastatic prostate cancer. The intervention was implemented alongside ADT with close longitudinal monitoring and was associated with favourable clinical, metabolic, body composition, and patient-reported outcomes during the observed follow-up period.

However, the single-case design and concurrent multimodal interventions preclude causal inference regarding the contribution of individual lifestyle components to the observed oncological outcomes. These findings should therefore be considered descriptive and hypothesis-generating and support further prospective investigation of structured, individualized lifestyle interventions as supportive strategies during ADT.

## Figures and Tables

**Table 1:** Changes in serum PSA and testosterone concentrations following lifestyle intervention and standard care.

Variables/Date	27/03/25	13/06/25	10/07/25	07/08/25	15/09/25	02/10/25	04/11/25	05/01/26	11/03/26	03/06/26
PSA (µg/L)	808.3	63.9	48.4.	16.9	7.6	6.2	4.6	2.5	1.7	1.7
Relative decrease (%)	-	92.1	24.1	65.1	55.3	18.0	26.1	46.0	32.0	0
Absolute decrease (%)	-	92.1	93.0	97.9	99.1	99.2	99.4	99.7	99.8	99.8
S-testosterone (nmol/L)	6.0	0.6	0.4	0.4	0.2	0.6	0.3	0.3	0.7	0.8

*Note.* PSA = prostate-specific antigen.

**Table 2:** Serial blood pressure monitoring during enzalutamide initiation.

Date	17/07/25	19/07/25	21/07/25	23/07/25	25/07/25	27/07/25	29/07/25	31/07/25	02/08/25	04/08/25	06/08/25
BP (mmHg)	116/72	116/73	110/65	111/68	108/67	108/66	115/67	110/65	111/70	112/68	111/71

*Note.* BP = blood pressure.

**Table 3:** Baseline and final metabolic, nutritional, hemodynamic, and body composition parameters.

Parameter	Baseline (27/03/2025)	Final (15/09/2025)
*Metabolic parameters*		
Total cholesterol (mmol/L)	4.7	4.6
HDL cholesterol (mmol/L)	1.9	1.6
LDL cholesterol (mmol/L)	2.4	2.6
Apolipoprotein B (mg/dL)	76.7	79.0
Triglycerides (mmol/L)	0.5	0.9
Fasting glucose	5.2	5.5
HbA1c (%)	5.4	4.9
HbA1C (mmol/mol)	35	30
*Hormonal parameters*		
Testosterone (µg/dL)	5.97	0.21
Cortisol (nmol/L)	312	412
*Hepatic parameters*		
ALT (U/L)	0.42	0.26
AST (U/L)	0.47	0.44
ALP (U/L)	1.30	1.67
γ-GT (U/L)	0.27	0.27
Total bilirubin (µmol/L)	17.3	10.5
Direct bilirubin (µmol/L)	6.0	3.7
*Renal parameters*		
Uric acid (µmol/L)	382	293
Creatinine (µmol/L)	77	52
eGFR (mL/min/1.73 m^2^)	>90	>90
*Haematological parameters*		
Haemoglobin (g/L)	148	141
Leucocytes (10^9^/L)	4.1	4.2
Lymphocytes (10^9^/L)	1.1	1.3
*Nutritional biomarkers*		
Vitamin B12 (pmol/L)	518	373
Folate (nmol/L)	35	36
25(OH)D (nmol/L)	134	221
Iron (μmol/L)	27	20
Ferritin (µg/L)	64	69
Homocysteine (µmol/L)	6.7	6.5
*Hemodynamic parameters*		
Blood pressure (mmHg)	116/72	111/71
*Body composition*		
Body mass (kg)	87.6	80.7
Body fat (%)	17.4	11.8
Fat-free mass (kg)	68.8	67.6
Fat-free mass (%)	78.5	83.8

*Note.* HDL cholesterol = high-density lipoprotein cholesterol. LDL cholesterol = low-density lipoprotein cholesterol. ALT = alanine aminotransferase. AST = aspartate aminotransferase. ALP = alkaline phosphatase. HbA1c = glycated hemoglobin. eGFR = estimated glomerular filtration rate.

**Table 4:** Sequential changes in body composition across FMD phases.

FMD (First Day)	30/04/25	1/06/25	25/06/25	10/08/25	02/10/25	21/11/25
BM (kg)						
Before	79.6	78.4	77.9	79.2	79.0	80.2
After	75.0	74.8	73.2	76.2	75.7	75.4
5 days after	79.2	78.3	77.8	77.4	78.8	79.6
BF (%)						
Before	13.9	11.3	9.7	9.3	12.0	11.3
After	13.5	10.2	9.1	10.9	10.7	10.9
5 days after	13.5	9.7	9.5	9.4	11.0	11.4
FFM (kg)						
Before	65.7	66.1	66.9	68.2	66.1	67.8
After	61.6	63.9	64.8	64.5	64.2	63.9
5 days after	65.6	67.2	66.9	66.7	66.6	67.1

*Note.* BM = body mass. BF = body fat. FFM = fat-free mass. FMD = fasting mimicking diet.

**Table 5:** Baseline and Intervention Dietary Intake.

Dietary Intake	Baseline–Omnivorous (Before 27/03/2025)	Intervention–WFLF Vegan (After 27/03/2025)
Energy (kcal)	2322	2690
Protein (g)	93	101
(% E)	16	15
Carbohydrate (g)	200	373
(% E)	34	55
Fiber (g)	35	75
Free sugar * (% E)	6	0
Fat (g)	120	72
(% E)	46	24
SFA (% E)	10	3
MUFA (% E)	17	8
PUFA (% E)	10	7
Cholesterol (mg)	280	0
Folate (µg)	343	1365
B12 (µg)	1.6	0
Vitamin C (mg)	70	172
Vitamin D (µg)	5.0	0
Calcium (mg)	754	975
Magnesium (mg)	513	1309
Potassium (mg)	1553	6011
Iron (mg)	18	38
Iodine (µg)	139	93
Zinc (mg)	11	15
Selenium (mg)	66	105
Water (l)	2.2	2.4
Alcohol (dl)	0	0

*Note.* Omnivorous = traditional mixed diet. WFLF vegan = whole-food, low-fat vegan diet. SFA = saturated fatty acids. MUFA = monounsaturated fatty acids. PUFA = polyunsaturated fatty acids. * All monosaccharides and disaccharides added to foods and beverages by the manufacturer, cook, or consumer, as well as sugars naturally present in honey, syrups, fruit juices, and fruit juice concentrates. % E = percentage of total energy intake. Standardized data and general Atwater energy conversion factors were applied (kcal/g): carbohydrates and protein 4, dietary fibre 2, fat 9, alcohol 7. Dietary supplements were not included in the dietary intake analysis. Dietary supplement use: the OMN diet included a multivitamin, whey protein shake, EPA/DHA omega-3, and aloe vera, whereas the VEG diet included vitamin B12, vitamin D3, EPA/DHA omega-3, a multivitamin, and a non-soy vegan protein shake.

## Data Availability

Copies of all original medical records and source documents have also been provided to the journal editor for verification and transparency purposes.
